# Intracortical inhibition, corticospinal excitability and voluntary activation in people with and without patellofemoral pain

**DOI:** 10.1113/EP093432

**Published:** 2026-02-16

**Authors:** Fawei Deng, Manali Akre, Aleksandra Birn‐Jeffery, Nelson Cortes, Jamie Tallent, Emily Finch, Xuerong Shao, Bernard Xian Wei Liew, Jia Han, Bradley Stephen Neal

**Affiliations:** ^1^ School of Sport, Rehabilitation and Exercise Sciences University of Essex Colchester UK; ^2^ Department of Bioengineering George Mason University Fairfax Virginia USA; ^3^ Department of Physiotherapy, Faculty of Medicine, Nursing and Health Science, School of Primary and Allied Health Care Monash University Melbourne Victoria Australia; ^4^ College of Rehabilitation Sciences Shanghai University of Medicine and Health Sciences Shanghai China; ^5^ Sports and Exercise Medicine Queen Mary University of London London UK

**Keywords:** patellofemoral pain, prognosis, reliability

## Abstract

The aims of this study were to investigate the intra‐rater reliability of peripheral nerve stimulation (PNS) and transcranial magnetic stimulation (TMS) in people with and without patellofemoral pain (PFP) and to compare nervous system function between these groups, using a case–control design. We sought people with and without PFP to participate in PNS and TMS testing to calculate maximal compound motor action potential, maximal force, voluntary activation (VA), active motor threshold (AMT), corticospinal excitability (CSE), silent period, and short‐interval intracortical inhibition. People with PFP also rated their current pain and function. Single‐measure intraclass correlation coefficients with 95% confidence intervals were used to determine intra‐rater reliability, with standard error of measurement and minimum detectable change calculated. Between‐group differences in PNS and TMS variables were determined using Student's two‐tailed, independent samples *t*‐tests or Mann–Whitney *U*‐tests. Twenty‐seven people without and 23 people with PFP completed PNS and TMS testing. For intra‐rater reliability, 18 people without and 17 people with PFP returned for a second testing session, and intraclass correlation coefficient values ranged from good to excellent (0.62–0.96). People with PFP demonstrated significantly lower VA (*P *< 0.0001), higher AMT (*P* = 0.014) and lower CSE (*P* = 0.018). In conclusion, both PNS and TMS demonstrate acceptable intra‐rater reliability in people with and without PFP. Elevated AMT and reduced CSE indicate that people with PFP might have a hypoexcitable motor cortex–corticospinal pathway, and lower VA indicates reduced recruitment of high‐threshold motor units. These findings indicate that a neurophysiological mechanism might underpin the poor prognosis of PFP.

## Abstract (highlights)

- **What is the central question of this study?**
  We investigated whether peripheral nerve stimulation and transcranial magnetic stimulation provide reliable neurophysiological measures in people with patellofemoral pain and whether these people show altered corticospinal excitability, intracortical inhibition, and voluntary activation compared with a control group.
- **What is the main finding and its importance?**
  Patellofemoral pain was associated with increased motor threshold, reduced corticospinal excitability, and lower voluntary activation, indicating impaired central motor drive. These findings identify a neurophysiological mechanism that might underlie the persistent symptoms and poor clinical outcomes in people with patellofemoral pain.

## 1 INTRODUCTION

Patellofemoral pain (PFP) is a common musculoskeletal condition experienced by people with varying levels of physical activity (Sterling et al., 2001). People with PFP report pain at, around or behind their patella that is aggravated by running, jumping, squatting, and stair ambulation (Crossley et al., 2016). PFP has a reported prevalence of 29% in active adolescents and 23% in the general population (Smith et al., 2018), with >50% of people reporting persistent pain 5–8 years postdiagnosis (Collins et al., 2013). Our previous meta‐regression identified that prolonged symptom duration was significantly associated with poor prognosis (Deng et al., 2025), highlighting the need to understand underlying mechanisms and optimal interventions.

Current understanding of PFP aetiology and intervention mechanisms has focused on biomotor factors, placing significant emphasis on the quadriceps muscles (Alsaleh et al., 2021; Lankhorst et al., 2016; Neal et al., 2019). People with PFP are reported to have weaker quadriceps prior to their symptom onset (Glaviano & Norte, 2022) that persists as their symptoms progress (Alsaleh et al., 2021; Lankhorst et al., 2016). Although knee‐targeted exercise therapy is considered best practice for improving pain and function in people with PFP (Neal et al., 2022, 2024), the associated underlying mechanisms of effect are poorly understood. Recent research has identified that altered neuromuscular control in people with PFP might be explained by altered primary motor cortex excitability (Te et al., 2017) as opposed to being related solely to biomotor factors (i.e., muscle weakness).

The combined use of peripheral nerve stimulation (PNS) and transcranial magnetic stimulation (TMS) provides a comprehensive approach to evaluating neuromuscular control from peripheral to cortical levels (Rossini et al., 2015). PNS is a non‐invasive technique used to evaluate voluntary activation (VA), which quantifies how effectively the brain activates skeletal muscles during active contraction (Ho et al., 2025). TMS is a non‐invasive neuromodulation technique that safely evaluates corticospinal excitability (CSE) (Rossini et al., 2015). Originally developed for the assessment and treatment of neuropsychiatric disorders, TMS has been used more recently in the investigation of persistent musculoskeletal conditions, including low‐back pain (Klein et al., 2015), knee osteoarthritis (Mansfield et al., 2022), and patellar tendinopathy (Vallance et al., 2024). This reflects the growing recognition of the potential role of neural adaptations in the persistence and modulation of musculoskeletal pain, underscoring the utility of PNS and TMS as non‐invasive assessment tools.

There is emerging evidence that people with PFP might demonstrate lower VA (Glaviano & Norte, 2022) and higher CSE (Parker et al., 2016) when compared with asymptomatic controls. It is plausible that subtle changes between intracortical neurones in the primary motor cortex (M1), corticospinal tract, and peripheral motor units might explain the poor prognosis experienced by people with PFP. To the best of our knowledge, nervous system function and the reliability of PNS/TMS has not been examined in people with or without PFP.

Our overarching aim was therefore to explore the role of the nervous system in explaining the motor dysfunction demonstrated by people with PFP. Specific objectives included: (1) determining the test–retest intra‐rater reliability of PNS/TMS in people with and without PFP; and (2) exploring whether nervous system function differs between people with and without PFP. We hypothesized that: (1) PNS/TMS will have acceptable reliability in people with and without PFP; and (2) people with PFP will have lower VA and higher CSE compared with people without PFP.

## 2 MATERIALS AND METHODS

### 2.1 Ethical approval

Ethical approval was obtained from the University of Essex Ethics Committee (ETH2324‐1373). All participants provided written informed consent before data collection. The study conformed to the standards of the latest revision of the *Declaration of Helsinki* but was not registered in a public trials database.

### 2.2 Sample size calculation

We calculated the required sample size using silent period (SP) data, the most frequently reported variable in other TMS studies involving people with musculoskeletal pain, given the lack of PFP‐specific literature. The effect size (Cohen's *d*) varied from 0.8 to 2.0 in publishd studies (On et al., 2004; Rio et al., 2016; Terada et al., 2016), and the smallest value (Cohen's *d*  =  0.8) was used to provide the most conservative estimate for between‐group difference. We required 21 participants per group to detect a similar between‐group difference in corticospinal inhibition with a β of 80% and an α of 5%. We therefore aimed to recruit 25 participants per group to allow for 20% data loss, and all recruited participants were invited to attend the laboratory for re‐test 48 h after their initial session.

### 2.3 Eligibility criteria

People aged from 18 to 45 years and of either biological sex were eligible to participate, provided they had no contraindications to safe participation in TMS/PNS data collection. People were eligible for the non‐PFP group provided they had no history of PFP and no current report of other musculoskeletal pain. People were eligible for the PFP group if they reported: (1) gradual onset of pain at, around or behind the patella; (2) symptoms provoked by at least two of the following activities: squatting, running, hopping/jumping, stair ascent/descent or prolonged sitting; and (3) symptoms for a minimum of 3 months, scored as ≥3/10 on a numerical pain rating scale (Deng et al., 2022). People were ineligible to participate in either group if they reported/demonstrated: (1) acute injury of the knee ligaments, joint capsule, bursa or meniscus; (2) knee‐extension or ‐flexion contracture deformity; (3) patellofemoral joint instability; (4) any traumatic, inflammatory or infectious disease of the lower limbs; and (5) a history of knee surgery.

### 2.4 Testing procedures

**FIGURE 1:**
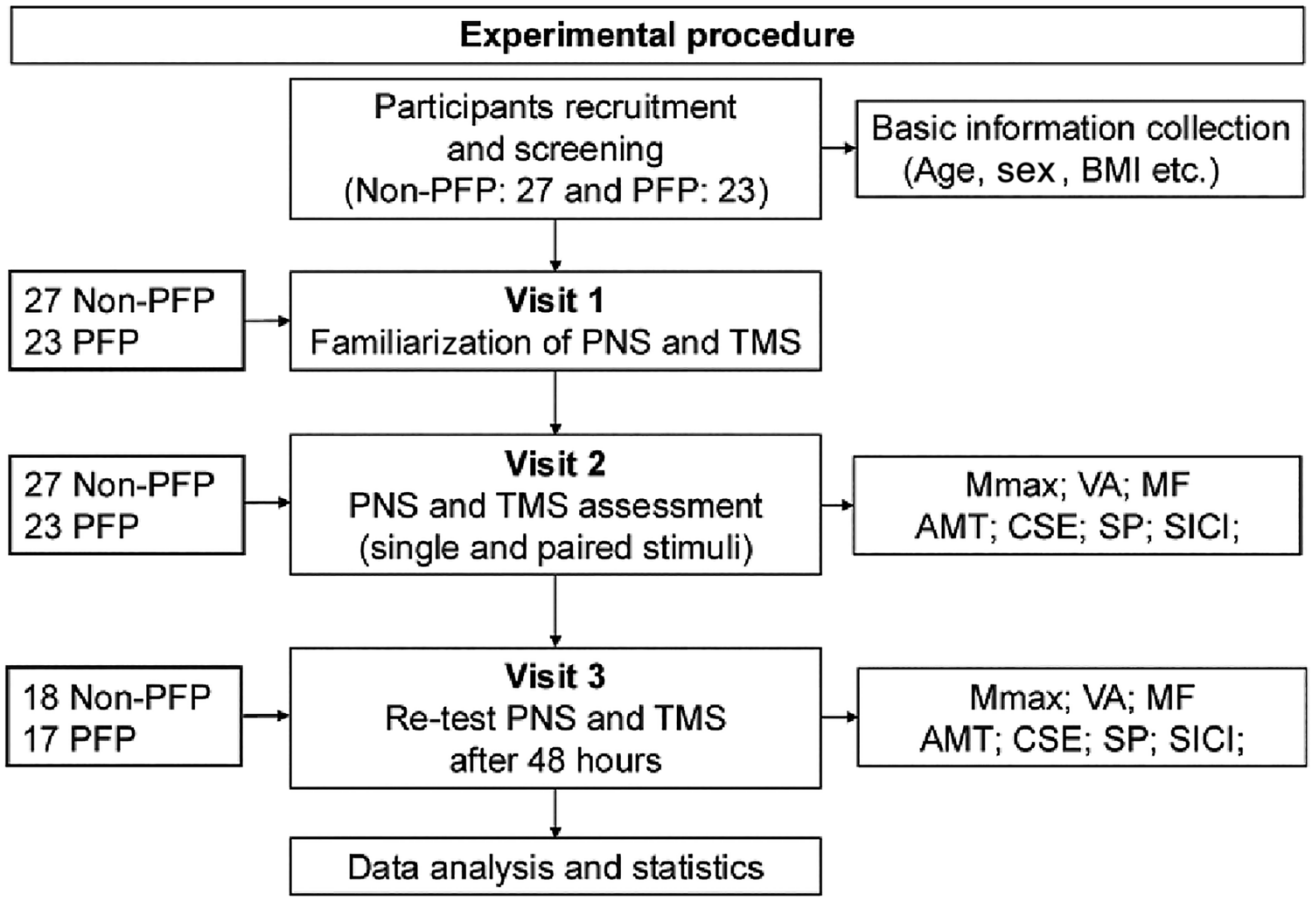
Experimental procedure. Abbreviations: AMT, active motor threshold; CSE, corticospinal excitability; MF, maximal force; *M*_max_, maximum compound muscle action potential; PFP, patellofemoral pain; PNS, peripheral nerve stimulation; SICI, short interval intracortical inhibition; SP, silent period; TMS—transcranial magnetic stimulation; VA, voluntary activation.

Both groups attended our laboratory a maximum of three times: first for familiarization and second for formal PNS and TMS data collection. Following this, willing participants returned for a third visit for exploration of reliability (see Figure 1). All neurophysiological measurements were obtained from the symptomatic limb in people with PFP (or the more symptomatic side if bilateral) and from the dominant limb in the control group.

We used surface electromyography (sEMG) to collect the muscle response during testing, with the rectus femoris as the targeted muscle. Initially, we abraded the targeted muscle belly using a single‐use disposable razor and sanitized the skin using a 75% alcohol disinfectant wipe to remove any material that could impede conductivity. Two surface electrodes were then placed 2 cm apart on the rectus femoris according to the Non‐Invasive Assessment of Muscles (SENIAM) guidelines (Hermens et al., 2000). An earth electrode was also placed over the centre of the patella. sEMG signals were amplified by a factor of 1000 using a digital amplifier (CED 1401, Cambridge Electronic Design, Cambridge, UK), bandpass filtered between 10 and 1000 Hz, and analysed off‐line (Signal v.6.06, Cambridge Electronic Design).

Seven variables were captured during data collection: maximal force (MF), maximum compound muscle action potential (*M*_max_), voluntary activation (VA), active motor threshold (AMT), corticospinal excitability (CSE), silent period (SP), and short interval intracortical inhibition (SICI).

#### 2.4.1 Quadriceps protocol

Before any data collection, participants readied their quadriceps for subsequent testing by performing three submaximal isometric knee‐extension contractions at 25%, 50%, and 75% of their perceived maximal effort, resting for 1 min between contractions. A calibrated load cell was used to measure maximal knee extensor force (in newtons). The load cell was fixed to a custom‐built chair and connected to a non‐compliant cuff placed around the participant's tibia, immediately superior to the ankle malleoli. Participants sat upright in the chair with their hips and knees at 90° flexion and were instructed to grasp the safety belt of the chair for support during contractions.

To quantify MF, participants were instructed to fold their arms across their chest and avoid any upper body movement, active hip flexion, or active ankle dorsiflexion. Participants were given verbal encouragement and visual feedback to help them reach MF. Each attempt lasted for 3 s and was followed by a 2 min rest period, and the highest sustained signal plateau was recorded as a participant's MF.

#### 2.4.2 PNS procedures

The *M*_max_ reflects the maximum electrical response of the targeted muscle when a superimposed peripheral nerve stimulation is given to a motor unit. *M*_max_ was collected using the superimposed burst technique (Rozand et al., 2015), which involves applying stimulation to the femoral nerve using a constant‐current stimulator (DS7AH, Digitimer Ltd, Welwyn Garden City, UK) with a pulse duration of 200 µs. This stimulation was delivered through two self‐adhesive surface electrodes (cathode position, femoral triangle; anode position, midway between the greater trochanter and iliac crest). PNS commenced with a single electrical stimulus to the resting muscle at 50 mA, and stimulus intensity was gradually increased by 20 mA until a plateau occurred. Superimposed stimulation was delivered by increasing the final stimulator output intensity by a further 30% (Rodriguez‐Falces & Place, 2018). The largest peak‐to‐peak sEMG response was recorded as a participant's *M*_max_ (Rozand et al., 2015).

VA reflects the ability of the CNS to activate a muscle fully during maximal voluntary contraction, reflecting excitability or inhibitory control within the brain (Glaviano & Norte, 2022). VA was collected using the twitch interpolation method (Rozand et al., 2015). A brief, high‐frequency burst of electrical stimulation was given to the femoral nerve while participants attempted a maximal isometric knee‐extension contraction. The stimulation intensity was matched to the intensity used to assess *M*_max_ (Glaviano & Norte, 2022). One electrical stimulus was applied immediately upon the plateau of maximal knee extensor force, and another stimulus was delivered after each MF (Rozand et al., 2015). Three measurements of VA were obtained and averaged, with a 2 min rest interval between contractions. The amplitude of the superimposed twitch delivered during maximal isometric knee‐extension contraction was compared with the amplitude of a resting, potentiated twitch delivered ∼2 s after the maximal isometric knee‐extension contraction. The following calculation was used to assess VA:

(1) $$\mathrm{VA}=1-\frac{\mathrm{superimposed}\;\mathrm{twitch}-\mathrm{force}\;\mathrm{stem}}{\mathrm{peripheral}\;\mathrm{twitch}}\times 100\%$$

#### 2.4.3 TMS procedures

The position of M1 was first identified using the Rossini–Rothwell method (Rothwell et al., 1999). The M1 area was stimulated using a magnetic stimulator (Magstim 2002, The Magstim Company Ltd, Whitland, UK) with a 110 mm double‐cone coil (D110 The Magstim Company Ltd). To induce a posterior–anterior cortical current and stimulate the hemisphere contralateral to the right lower limb, the coil was held and tilted to the side of the vertex by 1–2 cm. This area was used as the stimulation spot in all subsequent stimulations (Rothwell et al., 1999). TMS output intensity was first set at 30% and 40% intensity to familiarize the participant with the sensation. Subsequently, a consistent stimulator output of 50% was applied by adjusting the coil position until the greatest motor evoked potential (MEP) amplitude at the quadriceps was provoked.

After identifying M1, AMT, representing the minimum stimulus intensity required to elicit the MEP during a voluntary contraction, was determined while participants maintained 10% of their maximum knee‐extension force. The TMS output intensity was initially set at 50% and subsequently decreased until an MEP with a peak‐to‐peak amplitude exceeding 200 µV occurred. AMT was defined as the lowest intensity that elicited an MEP exceeding 200 µV at least three out of five times. A rest period of ≥5 s was implemented between each stimulation to prevent changes in cortical excitability, and this period was extended as needed based on participant comfort and tolerance (Rothwell et al., 1999).

CSE reflects the responsiveness of the pathway from the motor cortex to a peripheral muscle and is assessed by measuring the peak‐to‐peak amplitude of an MEP using single‐pulse TMS. Ten stimulations were applied to the M1 hotspot at 130% of AMT whilst participants actively performed knee extension with 10% of their maximal force. The stimulations were performed with a 15 s interstimulus interval. Peak‐to‐peak MEP amplitudes were measured in the rectus femoris muscle, recorded in millivolts, and normalized to *M*_max_ (MEP/*M*_max_ ratio). The average of the 10 trials was used for analysis. During the collection of CSE (130% AMT), SP was collected concurrently. SP was defined as the duration in milliseconds between MEP onset and the return of voluntary EMG activity via visual inspection, reflecting transient inhibition of corticospinal output (Hupfeld et al., 2020). To calculate CSE, a custom‐written code in the R programming language was used to extract the peak‐to‐peak amplitude. The duration of SP was collected from the stimulus artefact to the resumption of background EMG via visual inspection (Hupfeld et al., 2020).

SICI reflects the activity of GABA_A_ inhibitory interneurons within the motor cortex and was assessed using paired‐pulse TMS. Initially, one subthreshold conditioning stimulation (80% of AMT) was applied, followed by a suprathreshold test paired pulse (130% of AMT) with a 3 ms interstimulus interval (Neige et al., 2020). This paired‐pulse TMS was applied during 10% of participants’ maximal knee‐extension force and was repeated 10 times. To calculate SICI, we further normalized SICI to raw peak‐to‐peak amplitudes of the unconditioned MEP from single TMS stimulation. The SICI ratio (SICI/MEP) was selected to reflect the intracortical inhibition function. Both single‐ and paired‐pulse TMS procedures and intensities adhered to the most recent expert guidance (Rossi et al., 2021).

(2) $$\mathrm{SICI}\;\mathrm{ratio}=\frac{\mathrm{MEP}\;\mathrm{conditioned}}{\mathrm{MEP}\;\mathrm{unconditioned}\;}\times 100\%$$

#### 2.4.4 Patient‐reported outcome measures

A visual analog scale (VAS) and the patellofemoral subscale of the knee injury and osteoarthritis outcome score (KOOS‐PF) were used to record pain and function in people with PFP only (Hoglund et al., 2023). The VAS ranged from 0 (no pain at all) to 10 (worst pain imaginable), and the KOOS‐PF was scored from 0 to 100, with 100 representing no problems and 0 representing extreme problems. Average pain intensity (VAS‐A) and maximal pain intensity (VAS‐M) were recorded over the previous week, and pain intensity during the experiment (VAS‐E) and pain intensity during a maximal voluntary contraction (VAS‐MVC) were recorded.

### 2.5 Statistical analysis

All analyses were performed using the Statistical Package for the Social Sciences software program (v.26.0, IBM, Chicago, IL, USA). Single‐measure intraclass correlation coefficients (ICCs) with 95% confidence intervals were calculated using a two‐way mixed‐effects model with absolute agreement to determine intra‐rater reliability. ICCs were defined as excellent (>0.90), good (0.75–0.90), moderate (0.50–0.75) and poor (<0.50) (Koo & Li, 2016). Standard errors of measure (SEM) ($\mathrm{SD}\times \sqrt{1-\mathrm{ICC}})$ and minimum detectable change $\;(\mathrm{SEM}\times 1.96\times \sqrt{2}$) were also calculated (Shao et al., 2023). For between‐group analyses, the normality of distribution for all collected data was assessed using the Shapiro–Wilk test. Student's independent *t*‐tests were applied to normally distributed data, and Mann–Whitney tests were used for non‐normally distributed data.

## 3 RESULTS

### 3.1 Participant demographics

Twenty‐seven people without and 23 people with PFP were recruited. No differences were observed between groups for demographics or anthropometrics (Table 1).

**TABLE 1:** Descriptive characteristics.

Characteristic	Non‐PFP (*n* = 27)		PFP (*n* = 23)	
Sex, *n*	Male, 20	Female, 7	Male, 18	Female, 5
Age, years	23.6 ± 5.9	27.4 ± 4.4	24.8 ± 5.8	23.0 ± 5.2
Body mass index, kg/m^2^	24.0 ± 3.3	21.2 ± 3.1	24.2 ± 2.7	24.0 ± 2.8
Height, m	1.8 ± 0.0	1.6 ± 0.1	1.8 ± 0.1	1.7 ± 0.1
Weight, kg	76.6 ± 10.6	56.4 ± 7.6	76.3 ± 9.6	68.2 ± 12.3
Exercise frequency, per week	3.8 ± 1.4	3.3 ± 2.1	4.5 ± 1.5	3.8 ± 1.6
Symptom duration, months	–	–	25.3 ± 42.4	10.0 ± 6.0
KOOS‐PF	–	–	64.9 ± 18.8	65.5 ± 12.6
VAS‐A	–	–	3.6 ± 1.2	4.4 ± 0.9
VAS‐M	–	–	6.4 ± 1.7	7.6 ± 1.3
VAS‐E	–	–	0.7 ± 0.9	0.8 ± 0.8
VAS‐MVC	–	–	3.1 ± 2.1	3.0 ± 1.2

*Note*: Values are the mean ± SD. Abbreviations: KOOS‐PF, knee injury and osteoarthritis outcome score—patellofemoral subscale; MVC, maximum voluntary contraction; PFP, patellofemoral pain; VAS, visual analog scale.

### 3.2 PNS/TMS reliability

Reliability data were collected from 18 people without and 17 people with PFP. ICC values for both groups varied from moderate to excellent (0.62–0.96; Table 2).

**TABLE 2:** Reliability results from people without (*n *= 18) and people with (*n* = 17) patellofemoral pain.

Variable	Group	ICC	95% Confidence intervals	SD	Standard error of measurement	Minimum detectable change
*M*_max_, mV	Non‐PFP	0.66	0.29–0.86	1.47	0.86	2.37
	PFP	0.90	0.75–0.96	1.59	0.50	1.39
VA, %	Non‐PFP	0.73	0.42–0.89	0.06	0.03	0.08
	PFP	0.91	0.77–0.97	0.08	0.02	0.08
MF, N m/kg	Non‐PFP	0.90	0.75–0.96	1.28	0.36	0.99
	PFP	0.89	0.73–0.96	0.80	0.26	0.73
AMT, %MSO	Non‐PFP	0.84	0.62–0.94	5.92	2.37	6.57
	PFP	0.96	0.89–0.96	6.11	1.22	3.39
CSE, %*M*_max_	Non‐PFP	0.64	0.26–0.85	0.21	0.13	0.36
	PFP	0.62	0.22–0.84	0.14	0.08	0.24
SP, ms	Non‐PFP	0.83	0.60–0.93	0.02	0.01	0.02
	PFP	0.95	0.86–0.98	0.02	0.01	0.02
SICI ratio, %	Non‐PFP	0.71	0.37–0.88	0.21	0.12	0.32
	PFP	0.80	0.52–0.92	0.23	0.10	0.29

Abbreviations: AMT, active motor threshold; CSE, corticospinal excitability; ICC, intraclass correlation coefficient; MF, maximal force; *M*_max_, maximum compound muscle action potential; MSO, maximum stimulation output; PFP, patellofemoral pain; SICI, short interval intracortical inhibition; SP, silent period; VA, voluntary activation.

### 3.3 Between‐group analysis

**FIGURE 2:**
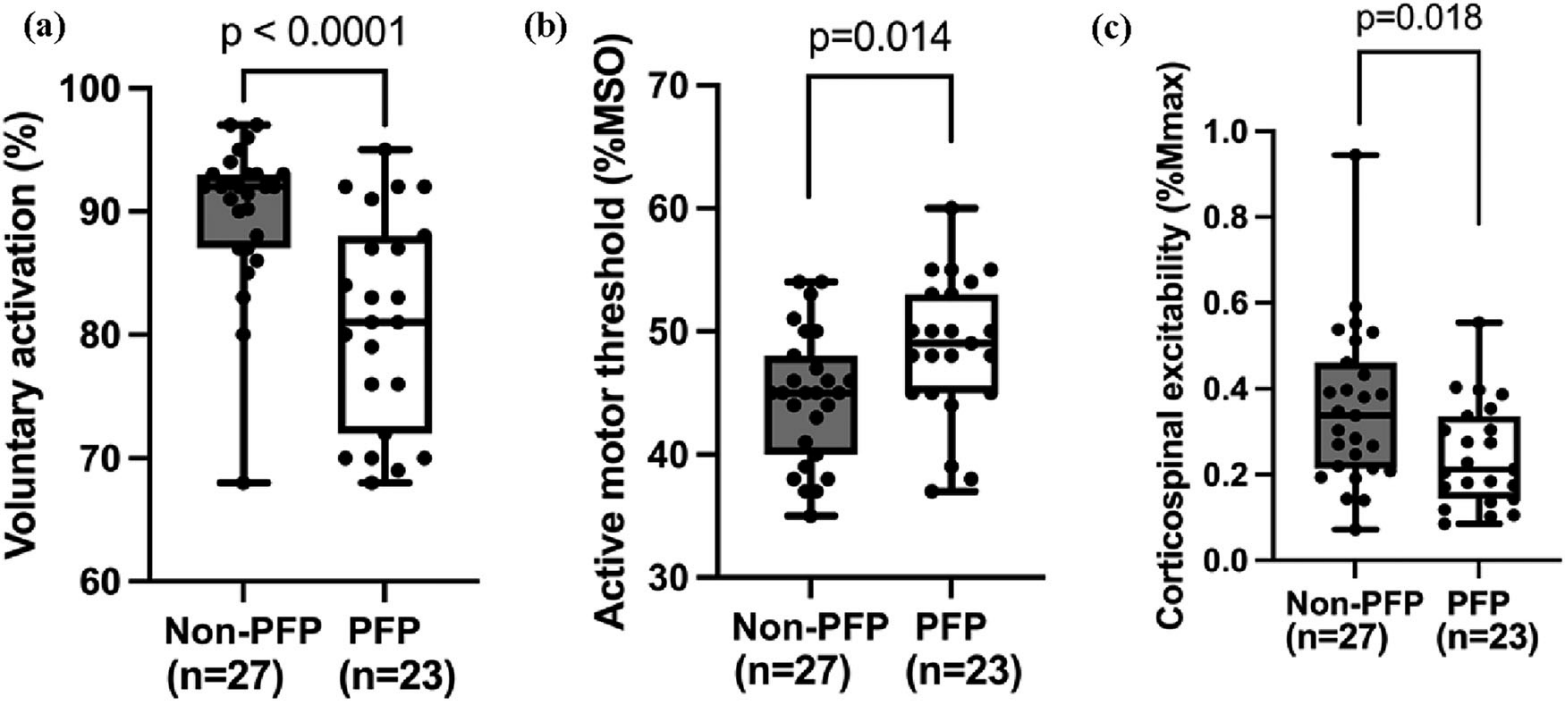
Group differences in voluntary activation (a), active motor threshold (b) and corticospinal excitability (c) between people with (*n* = 23) and without (*n* = 27) PFP. Abbreviations: *M*_max_, maximum compound muscle action potential; MSO, maximum stimulation output; PFP, patellofemoral pain.

**FIGURE A1:**
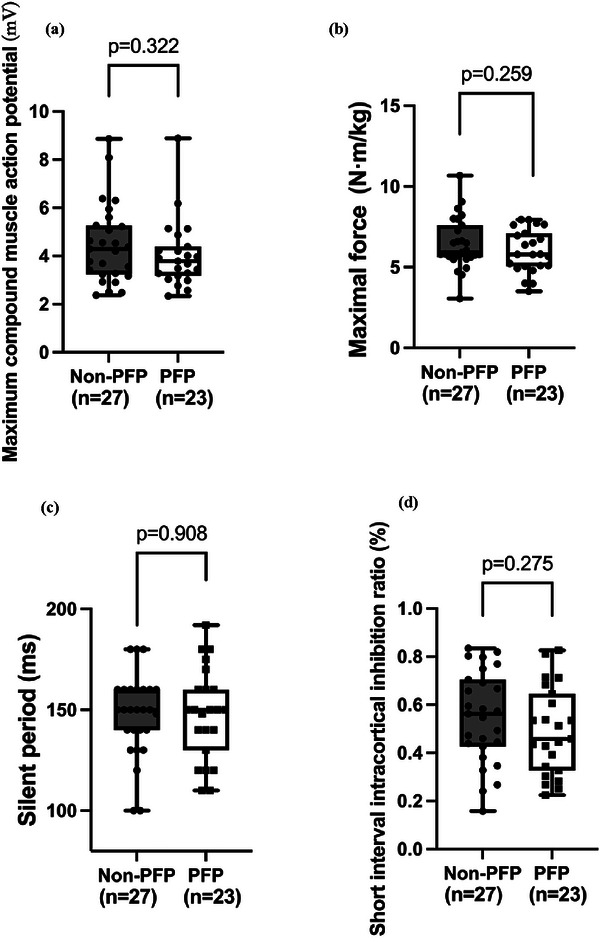
Group differences in maximum compound muscle action potential (a), maximal force (b), silent period (c) and short interval intracortical inhibition (d) ratio between people without (n = 27) and with (n = 23) patellofemoral pain (PFP).

People with PFP demonstrated significantly lower VA (*t* = 4.292; *P *< 0.0001; Cohen's *d* = 1.3; Figure 2a), higher AMT (*t* = −2.554; *P* = 0.014; Cohen's *d* = −0.7; Figure 2b) and lower CSE (*t* = 2.442; *P* = 0.018; Cohen's *d* = −0.7; Figure 2c). Detailed between‐group statistics are presented in Table 3. Analyses for all other variables were non‐significant (Appendix, Figure A1).

**TABLE 3:** Differences in nervous system function between people without (*n* = 27) and people with (*n* = 23) patellofemoral pain.

Parameter	Non‐PFP (*n* = 27)	PFP (*n* = 23)	Mean difference	95% Confidence intervals		*t*	*p*	Cohen's d
*M*_max_, mV	4.47 ± 1.63	4.03 ± 1.40	0.43	−0.44	1.31	1.00	0.322	0.28
VA, %	90.0 ± 6.0	81.1 ± 8.6	8.87	4.71	13.02	4.29	< 0.0001**	1.22
MF, N m/kg	6.45 ± 1.59	5.97 ± 1.33	0.48	−0.36	1.32	1.14	0.259	0.32
AMT, %MSO	45 ± 5	49 ± 6	−3.99	−7.14	−0.84	−2.54	0.014*	−0.72
CSE, %*M*_max_	35.41 ± 18.30	24.51 ± 12.02	10.90	1.92	19.88	2.44	0.018*	0.69
SP, ms	149 ± 19	149 ± 23	−0.5	−12.59	11.59	−0.08	0.908	−0.03
SICI ratio, %	54.85 ± 19.05	49.02 ± 18.11	5.83	−4.79	16.46	1.10	0.275	0.31

*Note*: Cohen's *d* effect sizes were interpreted as small (0.2), medium (0.5) and large (0.8) Values are presented as the mean ± SD. Abbreviations: AMT, active motor threshold; CSE, corticospinal excitability; MF, maximal force; *M*_max_, maximum compound muscle action potential; MSO, maximum stimulation output; PFP, patellofemoral pain; SICI, short interval intracortical inhibition; SP, silent period; VA, voluntary activation.

*P < 0.05 and **P < 0.001.

## 4 DISCUSSION

We aimed to evaluate the reliability of PNS/TMS measures in people with and without PFP. We identified moderate to excellent reliability (ICC range 0.62–0.96), supporting our hypothesis that both PNS/TMS are acceptable tools in people with and without PFP. Our findings partly supported our second hypothesis regarding altered neuromuscular function, in that we confirmed lower VA in people with PFP, but also lower CSE (rather than higher). The significantly lower VA observed in people with PFP indicates an impaired ability of the CNS to recruit motor units fully. The higher AMT and lower CSE observed in people with PFP suggest diminished corticospinal drive, which might impair effective muscle activation during functional tasks.

The lower VA that we identified in people with PFP is consistent with previous studies in this population (Ho et al., 2025; Kim et al., 2023), indicating an inability to recruit the quadriceps muscle fully. This aligns with the theory of arthrogenic muscle inhibition (AMI) (Ho et al., 2025; Rice & McNair, 2010), a reflex‐mediated suppression of muscle activation originating from a painful joint (Sherman et al., 2024). This suggests that people with PFP have reduced neural drive from their brain to their quadriceps, because the unaltered *M*_max_ and normalized MF (in newton metres per kilogram) that we identified indicates that peripheral neural excitability remains intact (Martucci et al., 2014). We also identified no differences in SICI ratio or SP, which indicates that the lower VA is not likely to be attributable to intracortical inhibition mediated by GABA_A_ or GABA_B_ (McDonnell et al., 2006). Prior research has reported conflicting outcomes regarding SP changes in chronic musculoskeletal conditions (Henry et al., 2011; Sherman et al., 2024), and our results further support that inhibition might not be affected. Our findings provide evidence that the neuromuscular dysfunction demonstrated by people with PFP might be centrally mediated and require the development of unique rehabilitation strategies.

Contrary to our hypothesis, we identified significantly higher AMT and lower CSE in people with PFP. Given that the corticospinal tract communicates the signal between the M1 and peripheral muscles (Rothwell et al., 1999), this diminished corticospinal drive might reveal the underlying mechanism of the motor dysfunction in people with PFP. Ho et al. (2022) also identified an elevated resting motor threshold in the vastus medialis and lateralis of people with PFP, and our findings concur despite using different methods and a different part of the quadriceps muscle. Comparable findings have also been reported in people with knee osteoarthritis and following anterior cruciate ligament injury (Sherman et al., 2024). On et al. (2004) have reported conflicting findings, with increased MEP amplitude of the vastus medialis in people with PFP, but when using raw MEP rather than MEP normalized to *M*_max_. The raw amplitude of MEP can be influenced by several methodological factors, including the placement of the electrodes and the position of the TMS coil, and normalizing to *M*_max_ is more robust (Rodriguez‐Falces & Place, 2018).

Although our primary findings highlight neurophysiological alterations at the cortical level (VA, AMT and CSE), these outcomes are likely to reflect both supraspinal and spinal‐level modulation. Previous studies have demonstrated reduced excitability of the vastus medialis H reflex in females with PFP, with lower H reflex amplitudes being strongly associated with greater pain, poorer function and longer symptom duration (de Oliveira Silva et al., 2017, 2016). These findings suggest that impaired A‐alpha afferent–motoneuron transmission and reflex inhibition might interact with cortical output to contribute to quadriceps dysfunction. This indicates that the AMI observed in our study is not solely from the cortical level but might also be from the pain‐induced spinal level. Given that this evidence comes from female samples and considering known sex differences in motor excitability and pain processing, including greater neuromuscular impairments in women (Kim et al., 2024), it is plausible that sex might influence both spinal and cortical adaptations. Accordingly, our findings should be interpreted with caution, given the sex imbalance in our sample, and further studies are required to explore potential sex‐related differences. From recent hypotheses regarding sensorimotor deficits, nociceptive inputs might trigger inhibitory pathways that suppress reflex excitability to protect the painful joint, thereby influencing the net corticospinal output we observed (Pazzinatto et al., 2017). Therefore, this interaction provides a plausible explanation for the concurrent spinal inhibition and cortical adaptations identified in PFP

By synthesizing the findings from both PNS and TMS, we have provided a more complete neurophysiological profile of people with PFP. The combination of lower VA and CSE, in the presence of preserved *M*_max_, indicates that the quadriceps weakness demonstrated by people with PFP is underpinned by central neural deficits. Although the reliability analysis indicated acceptable point estimates for most outcomes, several measures (e.g., CSE) showed wide ICC confidence intervals. This might reflect factors including inter‐individual variability, state‐dependent influences (e.g., fatigue) and the inherent sensitivity of neurophysiological measures. Consequently, the precision of these reliability estimates is relatively lower, and this should be considered when using these measures. Current gold‐standard interventions for PFP, including hip‐ and knee‐targeted exercise therapy, have shown success in improving symptoms and function in the short term at a population level, but do not benefit all. Our results suggest that some people with PFP might require more targeted, neurophysiologically informed interventions. Augmenting hip‐ and knee‐targeted exercise with metronome‐paced training has been demonstrated to upregulate motor cortex output in people without symptoms of knee pain (Akalu et al., 2025). Future studies should seek to explore whether motor cortex output in people with PFP can be improved with metronome‐paced training and whether this might be superior to traditional hip‐ and knee‐targeted exercise therapy. We acknowledge that the neurophysiological outcomes were tested without explicit correction for multiple comparisons. This decision was made to avoid the high risk of type II error associated with conservative corrections (e.g., Bonferroni) in this exploratory analysis, considering the physiological interdependence of these measures. Although the moderate effect sizes suggest meaningful physiological differences, the lack of correction raises the possibility of type I error; therefore, these findings should be interpreted with appropriate caution.

### 4.1 Strengths and limitations

To the best of our knowledge, this is the first study to assess comprehensively both corticospinal and intracortical excitability, in addition to voluntary muscle activation, in people with and without PFP. Establishing reliability of our neurophysiological techniques (PNS and TMS) adds rigour. In comparison to previous studies exploring nervous system function in people with PFP, our sample size is larger and was defined a priori, reducing the potential for error.

One limitation is the balance of male and female participants, with females participants making up merely 24% of our total sample. This was not intentional, but it does reduce the external applicability of our findings, and future studies should seek to confirm whether our findings also apply to females with PFP. Symptom duration was prolonged and heterogeneous in our PFP population, which represents a limitation common in PFP studies (Briani et al., 2025; Collins et al., 2010). Variability in motoneuron pool excitability, state‐dependent influences (e.g., fatigue) and the use of *M*_max_ for normalization might all introduce noise into CSE estimates. This is reflected in the moderate reliability observed for CSE in the PFP group (ICC = 0.62), indicating greater measurement variability than for other outcomes. Although the between‐group differences remain, these limitations suggest that conclusions regarding corticospinal excitability should be drawn cautiously.

## 5 CONCLUSION

This study provides new evidence of decreased voluntary activation and reduced corticospinal excitability in people with PFP and shows that the tools used to measure these variables are reliable. Diminished corticospinal drive and impaired central motor output might be part of the mechanism underlying the poor prognosis experienced by some people with PFP. These findings underscore the importance of CNS adaptations in managing people with PFP and that interventions might need to be designed to address these deficits specifically.

## AUTHOR CONTRIBUTIONS

Fawei Deng: Conceptualization, Methodology, Investigation, Data curation, Formal analysis, Visualization, Writing—original draft, Writing—review & editing. Manali Akre: Conceptualization, Methodology, Investigation, Data curation, Writing—review & editing. Aleksandra Birn‐Jeffery: Conceptualization, Methodology, Visualization, Supervision, Writing—review & editing. Cortes Nelson: Conceptualization, Methodology, Visualization, Supervision, Writing—review & editing. Jamie Tallent: Conceptualization, Methodology, Software, Writing—review & editing. Emily Finch: Conceptualization, Methodology, Software, Writing—review & editing. Xuerong Shao: Formal Analysis, Writing—Review & Editing Bernard Liew: Formal Analysis, Writing—Review & EditingJia Han: Formal Analysis, Writing—Review & Editing. Bradley Neal: Conceptualization, Methodology, Investigation, Data curation, Formal analysis, Visualization, Supervision, Writing—review & editing. All authors approved the final version of the manuscript and agree to be accountable for all aspects of the work in ensuring that questions related to the accuracy or integrity of any part of the work are appropriately investigated and resolved. All persons designated as authors qualify for authorship, and all those who qualify for authorship are listed.

## CONFLICT OF INTEREST

None declared.

## Supporting information



Supporting Information

## Data Availability

The data supporting the findings of this study are available as Supporting information for online publication.

## References

[eph70228-bib-0001] Akalu, Y. , Tallent, J. , Frazer, A. K. , Siddique, U. , Rostami, M. , Howatson, G. , Walker, S. , & Kidgell, D. J (2025). Determining the cortical, corticospinal, and reticulospinal responses to metronome‐paced and self‐paced strength training. European Journal of Applied Physiology, 1–24. 10.1007/s00421-025-05939-3 PMC13013172 40886203

[eph70228-bib-0002] Alsaleh, S. A. , Murphy, N. A. , Miller, S. C. , Morrissey, D. , & Lack, S. D (2021). Local neuromuscular characteristics associated with patellofemoral pain: A systematic review and meta‐analysis. Clinical Biomechanics (Bristol, Avon), 90, 105509. 34678670 10.1016/j.clinbiomech.2021.105509

[eph70228-bib-0003] Briani, R. V. , Balotari, A. F. B. , Waiteman, M. C. , Magalhães, F. H. , Bazett‐Jones, D. M. , & de Azevedo, F. M. (2025). Is self‐reported symptom duration in individuals with patellofemoral pain an accurate measure? An observational longitudinal study. Brazilian Journal of Physical Therapy, 29(1), 101167. 39742736 10.1016/j.bjpt.2024.101167 PMC11750445

[eph70228-bib-0004] Collins, N. J. , Bierma‐Zeinstra, S. M. , Crossley, K. M. , van Linschoten, R. L. , Vicenzino, B. , & van Middelkoop, M. (2013). Prognostic factors for patellofemoral pain: A multicentre observational analysis. British Journal of Sports Medicine, 47(4), 227–233. 23242955 10.1136/bjsports-2012-091696

[eph70228-bib-0005] Collins, N. J. , Crossley, K. M. , Darnell, R. , & Vicenzino, B. (2010). Predictors of short and long term outcome in patellofemoral pain syndrome: A prospective longitudinal study. BMC Musculoskeletal Disorders [Electronic Resource], 11(1), 11. 20082723 10.1186/1471-2474-11-11 PMC2823664

[eph70228-bib-0006] Crossley, K. M. , Stefanik, J. J. , Selfe, J. , Collins, N. J. , Davis, I. S. , Powers, C. M. , McConnell, J. , Vicenzino, B. , Bazett‐Jones, D. M. , Esculier, J. F. , Morrissey, D. , & Callaghan, M. J. (2016). 2016 Patellofemoral pain consensus statement from the 4th International Patellofemoral Pain Research Retreat, Manchester. Part 1: Terminology, definitions, clinical examination, natural history, patellofemoral osteoarthritis and patient‐reported outcome measures. British Journal of Sports Medicine, 50(14), 839–843. 27343241 10.1136/bjsports-2016-096384 PMC4975817

[eph70228-bib-0007] Deng, F. , Adams, R. , Pranata, A. , Cui, F. , & Han, J. (2022). Tibial internal and external rotation taping for improving pain in patients with patellofemoral pain syndrome. Journal of Science and Medicine in Sport, 25(8), 644–648. 35484009 10.1016/j.jsams.2022.04.003

[eph70228-bib-0008] Deng, F. , Razaviasfali, S. M. , Birn‐Jeffery, A. , Cortes, N. , & Neal, B. S. (2025). What prognostic indicators and treatment mechanisms exist for efficacious treatments in people with patellofemoral pain? A secondary meta‐regression with an updated search. JOSPT Open, 3(2), 193–209.

[eph70228-bib-0009] de Oliveira Silva, D. , Magalhães, F. H. , Faria, N. C. , Ferrari, D. , Pazzinatto, M. F. , Pappas, E. , & de Azevedo, F. M. (2017). Vastus medialis hoffmann reflex excitability is associated with pain level, self‐reported function, and chronicity in women with patellofemoral pain. Archives of Physical Medicine and Rehabilitation, 98(1), 114–119. 27422350 10.1016/j.apmr.2016.06.011

[eph70228-bib-0010] de Oliveira Silva, D. , Magalhães, F. H. , Faria, N. C. , Pazzinatto, M. F. , Ferrari, D. , Pappas, E. , & de Azevedo, F. M. (2016). Lower amplitude of the Hoffmann reflex in women with patellofemoral pain: Thinking beyond proximal, local, and distal factors. Archives of Physical Medicine and Rehabilitation, 97(7), 1115–1120. 26763946 10.1016/j.apmr.2015.12.017

[eph70228-bib-0011] Glaviano, N. R. , & Norte, G. E. (2022). Gluteal central activation in females with patellofemoral pain: A preliminary study. Journal of Sport Rehabilitation, 31(6), 676–683. 34883467 10.1123/jsr.2021-0093

[eph70228-bib-0012] Henry, D. E. , Chiodo, A. E. , & Yang, W. (2011). Central nervous system reorganization in a variety of chronic pain states: A review. Physical Medicine and Rehabilitation, 3(12), 1116–1125. 10.1016/j.pmrj.2011.05.018 22192321

[eph70228-bib-0013] Hermens, H. J. , Freriks, B. , Disselhorst‐Klug, C. , & Rau, G. (2000). Development of recommendations for SEMG sensors and sensor placement procedures. Journal of Electromyography and Kinesiology, 10(5), 361–374. 11018445 10.1016/s1050-6411(00)00027-4

[eph70228-bib-0014] Ho, K. Y. , Carpio, M. , Donohue, J. , Kissman, J. , & Liang, J. N. (2025). Comparison of gluteal muscle central activation in individuals with and without patellofemoral pain. Frontiers in Physiology, 16, 1535141. 40041163 10.3389/fphys.2025.1535141 PMC11876401

[eph70228-bib-0015] Ho, K. Y. , Liang, J. N. , Budge, S. , Madriaga, A. , Meske, K. , & Nguyenton, D. (2022). Brain and Spinal Cord Adaptations Associated With Patellofemoral Pain: A Systematic Review and Meta‐Analysis. Frontiers in Integrative Neuroscience, 16, 791719. 35197832 10.3389/fnint.2022.791719 PMC8859985

[eph70228-bib-0016] Hoglund, L. T. , Scalzitti, D. A. , Bolgla, L. A. , Jayaseelan, D. J. , & Wainwright, S. F. (2023). Patient‐reported outcome measures for adults and adolescents with patellofemoral pain: A systematic review of content validity and feasibility using the COSMIN methodology. Journal of Orthopaedic and Sports Physical Therapy, 53(1), 23–39. 36251651 10.2519/jospt.2022.11317

[eph70228-bib-0017] Hupfeld, K. E. , Swanson, C. W. , Fling, B. W. , & Seidler, R. D. (2020). TMS‐induced silent periods: A review of methods and call for consistency. Journal of Neuroscience Methods, 346, 108950. 32971133 10.1016/j.jneumeth.2020.108950 PMC8276277

[eph70228-bib-0018] Kim, S. , Glaviano, N. R. , & Park, J. (2024). Sex differences in knee extensor neuromuscular function in individuals with and without patellofemoral pain. Sports Health, 16(6), 1000–1008. 37978417 10.1177/19417381231209318 PMC11531066

[eph70228-bib-0019] Kim, S. , Roh, Y. , Glaviano, N. R. , & Park, J. (2023). Quadriceps neuromuscular function during and after exercise‐induced fatigue in patients with patellofemoral pain. Journal of Athletic Training, 58(6), 554–562. 36395370 10.4085/1062-6050-0348.22 PMC10496447

[eph70228-bib-0020] Klein, M. M. , Treister, R. , Raij, T. , Pascual‐Leone, A. , Park, L. , Nurmikko, T. , Lenz, F. , Lefaucheur, J. P. , Lang, M. , Hallett, M. , Fox, M. , Cudkowicz, M. , Costello, A. , Carr, D. B. , Ayache, S. S. , & Oaklander, A. L. (2015). Transcranial magnetic stimulation of the brain: Guidelines for pain treatment research. Pain, 156(9), 1601–1614. 25919472 10.1097/j.pain.0000000000000210 PMC4545735

[eph70228-bib-0021] Koo, T. K. , & Li, M. Y. (2016). A guideline of selecting and reporting intraclass correlation coefficients for reliability research. Journal of Chiropractic Medicine, 15(2), 155–163. 27330520 10.1016/j.jcm.2016.02.012 PMC4913118

[eph70228-bib-0022] Lankhorst, N. E. , van Middelkoop, M. , Crossley, K. M. , Bierma‐Zeinstra, S. M. , Oei, E. H. , Vicenzino, B. , & Collins, N. J. (2016). Factors that predict a poor outcome 5–8 years after the diagnosis of patellofemoral pain: A multicentre observational analysis. British Journal of Sports Medicine, 50(14), 881–886. 26463119 10.1136/bjsports-2015-094664

[eph70228-bib-0023] Mansfield, C. J. , Culiver, A. , Briggs, M. , Schmitt, L. C. , Grooms, D. R. , & Oñate, J. (2022). The effects of knee osteoarthritis on neural activity during a motor task: A scoping systematic review. Gait & Posture, 96, 221–235. 35700640 10.1016/j.gaitpost.2022.05.035

[eph70228-bib-0024] Martucci, K. T. , Ng, P. , & Mackey, S. (2014). Neuroimaging chronic pain: What have we learned and where are we going? Future Neurology, 9(6), 615–626. 28163658 10.2217/FNL.14.57 PMC5289824

[eph70228-bib-0025] McDonnell, M. N. , Orekhov, Y. , & Ziemann, U. (2006). The role of GABA(B) receptors in intracortical inhibition in the human motor cortex. Experimental Brain Research, 173(1), 86–93. 16489434 10.1007/s00221-006-0365-2

[eph70228-bib-0026] Neal, B. S. , Bartholomew, C. , Barton, C. J. , Morrissey, D. , & Lack, S. D. (2022). Six treatments have positive effects at 3 months for people with patellofemoral pain: A systematic review with meta‐analysis. Journal of Orthopaedic and Sports Physical Therapy, 52(11), 750–768. 36070427 10.2519/jospt.2022.11359

[eph70228-bib-0027] Neal, B. S. , Lack, S. D. , Bartholomew, C. , & Morrissey, D. (2024). Best practice guide for patellofemoral pain based on synthesis of a systematic review, the patient voice and expert clinical reasoning. British Journal of Sports Medicine, 58(24), 1486–1495. 39401870 10.1136/bjsports-2024-108110

[eph70228-bib-0028] Neal, B. S. , Lack, S. D. , Lankhorst, N. E. , Raye, A. , Morrissey, D. , & van Middelkoop, M. (2019). Risk factors for patellofemoral pain: A systematic review and meta‐analysis. British Journal of Sports Medicine, 53(5), 270–281. 30242107 10.1136/bjsports-2017-098890

[eph70228-bib-0029] Neige, C. , Grospretre, S. , Martin, A. , & Lebon, F. (2020). Influence of voluntary contraction level, test stimulus intensity and normalization procedures on the evaluation of short‐interval intracortical inhibition. Brain Sciences, 10(7), 433. 32650395 10.3390/brainsci10070433 PMC7407177

[eph70228-bib-0030] On, A. Y. , Uludağ, B. , Taşkiran, E. , & Ertekin, C. (2004). Differential corticomotor control of a muscle adjacent to a painful joint. Neurorehabilitation and Neural Repair, 18(3), 127–133. 15375272 10.1177/0888439004269030

[eph70228-bib-0031] Parker, R. S. , Lewis, G. N. , Rice, D. A. , & McNair, P. J. (2016). Is motor cortical excitability altered in people with chronic pain? A systematic review and meta‐analysis. Brain Stimulation, 9(4), 488–500. 27133804 10.1016/j.brs.2016.03.020

[eph70228-bib-0032] Pazzinatto, M. F. , de Oliveira Silva, D. , Pappas, E. , Magalhães, F. H. , & de Azevedo, F. M. (2017). Is quadriceps H‐reflex excitability a risk factor for patellofemoral pain? Medical Hypotheses, 108, 124–127. 29055385 10.1016/j.mehy.2017.08.019

[eph70228-bib-0033] Rice, D. A. , & McNair, P. J. (2010). Quadriceps arthrogenic muscle inhibition: Neural mechanisms and treatment perspectives. Seminars in Arthritis and Rheumatism, 40(3), 250–266. 19954822 10.1016/j.semarthrit.2009.10.001

[eph70228-bib-0034] Rio, E. , Kidgell, D. , Moseley, G. L. , & Cook, J. (2016). Elevated corticospinal excitability in patellar tendinopathy compared with other anterior knee pain or no pain. Scandinavian Journal of Medicine & Science in Sports, 26(9), 1072–1079. 26369282 10.1111/sms.12538

[eph70228-bib-0035] Rodriguez‐Falces, J. , & Place, N. (2018). Determinants, analysis and interpretation of the muscle compound action potential (M wave) in humans: Implications for the study of muscle fatigue. European Journal of Applied Physiology, 118(3), 501–521. 29282530 10.1007/s00421-017-3788-5

[eph70228-bib-0036] Rossi, S. , Antal, A. , Bestmann, S. , Bikson, M. , Brewer, C. , Brockmöller, J. , Carpenter, L. L. , Cincotta, M. , Chen, R. , Daskalakis, J. D. , Di Lazzaro, V. , Fox, M. D. , George, M. S. , Gilbert, D. , Kimiskidis, V. K. , Koch, G. , Ilmoniemi, R. J. , Lefaucheur, J. P. , Leocani, L. , …, Hallett, M. (2021). Safety and recommendations for TMS use in healthy subjects and patient populations, with updates on training, ethical and regulatory issues: Expert guidelines. Clinical Neurophysiology, 132(1), 269–306. 33243615 10.1016/j.clinph.2020.10.003 PMC9094636

[eph70228-bib-0037] Rossini, P. M. , Burke, D. , Chen, R. , Cohen, L. G. , Daskalakis, Z. , Di Iorio, R. , Di Lazzaro, V. , Ferreri, F. , Fitzgerald, P. B. , George, M. S. , Hallett, M. , Lefaucheur, J. P. , Langguth, B. , Matsumoto, H. , Miniussi, C. , Nitsche, M. A. , Pascual‐Leone, A. , Paulus, W. , Rossi, S. , …, Ziemann, U. (2015). Non‐invasive electrical and magnetic stimulation of the brain, spinal cord, roots and peripheral nerves: Basic principles and procedures for routine clinical and research application. An updated report from an I.F.C.N. Committee. Clinical Neurophysiology, 126(6), 1071–1107. 25797650 10.1016/j.clinph.2015.02.001 PMC6350257

[eph70228-bib-0038] Rothwell, J. C. , Hallett, M. , Berardelli, A. , Eisen, A. , Rossini, P. , & Paulus, W. (1999). Magnetic stimulation: Motor evoked potentials. The International Federation of Clinical Neurophysiology. Electroencephalography and clinical neurophysiology. Supplement, 52, 97–103. 10590980

[eph70228-bib-0039] Rozand, V. , Grosprêtre, S. , Stapley, P. J. , & Lepers, R. (2015). Assessment of neuromuscular function using percutaneous electrical nerve stimulation. Journal of visualized experiments: JoVE, 00(103), 52974. 10.3791/52974 PMC4692605 26436986

[eph70228-bib-0040] Shao, X. , Kang, M. , Luan, L. , Deng, F. , Adams, R. , Wu, T. , & Han, J. (2023). Reliability and validity of the ankle inversion discrimination apparatus during walking in individuals with chronic ankle instability. Frontiers in Physiology, 14, 1036194. 36744024 10.3389/fphys.2023.1036194 PMC9893012

[eph70228-bib-0041] Sherman, D. A. , Rush, J. , Glaviano, N. R. , & Norte, G. E. (2024). Knee joint pathology and efferent pathway dysfunction: Mapping muscle inhibition from motor cortex to muscle force. Musculoskeletal Science and Practice, 74, 103204. 39426249 10.1016/j.msksp.2024.103204

[eph70228-bib-0042] Smith, B. E. , Selfe, J. , Thacker, D. , Hendrick, P. , Bateman, M. , Moffatt, F. , Rathleff, M. S. , Smith, T. O. , & Logan, P. (2018). Incidence and prevalence of patellofemoral pain: A systematic review and meta‐analysis. PLoS ONE, 13(1), e0190892. 29324820 10.1371/journal.pone.0190892 PMC5764329

[eph70228-bib-0043] Sterling, M. , Jull, G. , & Wright, A. (2001). The effect of musculoskeletal pain on motor activity and control. The Journal of Pain, 2(3), 135–145. 14622823 10.1054/jpai.2001.19951

[eph70228-bib-0044] Te, M. , Baptista, A. F. , Chipchase, L. S. , & Schabrun, S. M. (2017). Primary Motor Cortex Organization Is Altered in Persistent Patellofemoral Pain. Pain Medicine (Malden, Mass.), 18(11), 2224–2234. 28340134 10.1093/pm/pnx036

[eph70228-bib-0045] Terada, M. , Bowker, S. , Thomas, A. C. , Pietrosimone, B. , Hiller, C. E. , & Gribble, P. A. (2016). Corticospinal excitability and inhibition of the soleus in individuals with chronic ankle instability. Physical Medicine and Rehabilitation, 8(11), 1090–1096. 10.1016/j.pmrj.2016.04.006 27208398

[eph70228-bib-0046] Vallance, P. , Malliaras, P. , Vicenzino, B. , & Kidgell, D. J. (2024). Determining intracortical, corticospinal and alpha motoneurone excitability in athletes with patellar tendinopathy compared to asymptomatic controls. Scandinavian Journal of Medicine & Science in Sports, 34(2), e14579. 38332685 10.1111/sms.14579

